# A Curious War Incident

**Published:** 1892-02

**Authors:** 


### A CURIOUS WAR INCIDENT.

An officer of the historical 5th New Hampshire regiment, related to us the following strange coincidence, which at our request he formulated for publication in our columns:

The golden fields of grain and the pretty villages of Maryland presented a striking contrast to the dismal swamps and muddy streams of the peninsula, and our men marched willingly on to the field of Antietam, my regiment being, as usual, on the skirmish line.

It was a lovely morning, that 14th of September, 1862, and we moved rapidly on through the towns of Boonsboro', Keedysville, and Tunleytown, and found the enemy occupying a position beyond the Antietam river. The confederate artillery opened on our lines as they hove in sight, from points on the high ground on the west side of the stream. On reaching the river we were ordered to halt, and a few moments later the roar of Hooker's guns was heard as the blue line of his infantry was seen dashing down the low ground on our left; then the sharp crack of the rifle ten-pounders, and the steady rattle of musketry; the brave cheer of our men, and the yell of the rebels, all came upon us. As we stood gazing upon the scene our colonel rode past and said: "Let not a man leave the line, be ready to move at a moment's notice: we are to support Gen. Hooker."

But I did not set out in writing this brief story to rehearse the events of the Battle of Antietam, which are doubtless familiar to all old soldiers and readers of current history. Let me return to the village of Tunleytown, where is laid the scene of this hasty sketch.

As I before stated, the 5th regiment was doing skirmish duty, and in passing on through the village Company K had to file through a lane over fences and between a number of out-buildings. Capt. C., in order to make a short cut and regain his company, which was deployed, passed through a house and came upon a woman frying doughnuts in a kettle over an open fire place, and upon the floor a child, about two years old, was playing. The woman in attendance was somewhat startled at the sight of the big dusty looking soldier, and held up her hands, exclaiming, "My Lord, soldiers! where did you Lincoln men come from?" Capt. C. had no time to give her the required information and merely said, "Yes, Lincoln soldiers. There will be a big battle near here to-morrow; you had better be getting ready to move away." "Will you take some of these hot cakes with you?" she asked. "Yes, with pleasure," said Capt. C. She filled a small bag with the hot fried cakes (the like of which the captain had not seen since he left the old Granite State) and wished him God speed and victory. Now, as Capt. C. had no scrip, nor any thing which had a "metallic ring" about him save a McClellan medal of brass, about the size of a half-dollar, with the impress of Gen. McClellan on one side and on the other the name of the officer and the regiment to which he belonged, carried by many of our soldiers for identification in case of accident, the captain, thanked the lady for her kindness and liberality, gave his McClellan medal to the child, hanging it around its neck and passed on to join his company. Nothing more was thought of the matter by the captain, who served with his regiment till the close of the war, and then sought other pursuits. Twenty-eight years passed and Capt. C. found himself in Washington. In walking out one morning on Pennsylvania avenue, he was accosted by an elderly gentleman who was in a terribly excited state of mind about his wife and daughter, whom he had lost, he said: "We came in on the train from Florida last night where we had been spending the winter, and I told my wife and daughter they could go and look at the Capitol and be back by 7 o'clock. They have been absent all night. I have been to the police station and made enquiries everywhere for them, but cannot hear a word from them. O, dear, I reckon they are lost." The old gentleman told the captain he was from Tunleytown, Md., and had lived there for forty years. While they were talking two women hove in sight, one an elderly person, the other much younger. "There they are now! dod rot it, where did they come from?"

A general explanation followed. It seemed the ladies went to the railroad station after visiting the Capitol and had remained there all the time waiting for his lordship to put in an appearance, and he had searched *everywhere* else for them. The old gentleman introduced the captain to his wife and daughter and invited him to call on him if he ever visited Tunleytown. "Tunleytown, Tunleytown," said the captain, "is not that village near the battlefield of Antietam?" "Why, yes," said the old lady. "Well," replied the whilom captain, "I should like to know if there is a lady living there now who filled my haversack with doughnuts the day before the battle?" "Yes, sir, she is, and there she stands, and *here is her medal!*" Sure enough there stood a beautiful lady who had around her neck on a gold chain my old McClellan medal, which she had kept and worn ever since the morning of the 14th of September, 1862. A general conversation followed, in which the old gentleman and his wife related all the circumstances as I have done to the reader, and added that they read over the list of killed at the Battle of Antietam, but did not see my name among them, but later on they had seen one of my name from the same regiment, who was killed at Gettysburg. It is needless to say that the captain was not loth to accept a very pressing invitation to go to Tunleytown and have some more doughnuts when *not* in so much of a hurry.

